# An Integrated Approach to the Sustainable Remediation of Dairy Industry Effluents: Sequential Processes of Coagulation–Flocculation, Adsorption, and Photocatalysis

**DOI:** 10.1021/acsomega.6c05070

**Published:** 2026-08-07

**Authors:** Luiz Eduardo Nochi Castro, Larissa Resende Matheus, Maycon Diego Ribeiro, Leda Maria Saragiotto Colpini

**Affiliations:** Advanced Campus of Jandaia do Sul, Federal University of Paraná (UFPR), Rua Dr João Maximiano, 426, Centro, 86900-000 Jandaia do Sul, Paraná, Brazil

## Abstract

Cheese whey wastewater (CWW) is a highly polluting effluent characterized by elevated organic load (COD ≈ 71,000 mg L^–1^) and turbidity (≈3250 NTU). In this study, a sequential treatment system combining coagulation/flocculation, adsorption, and heterogeneous photocatalysis was applied for CWW remediation. Coagulation/flocculation using an anionic polyacrylamide achieved removals of ∼22% COD, ∼37% BOD_5_, ∼30% turbidity, and >80% suspended solids. Subsequent adsorption with activated carbon increased removal efficiency to ∼65% COD, ∼55% BOD_5_, and ∼60% turbidity. Activated carbon characterization confirmed a porous structure and functional groups responsible for adsorption. The final TiO_2_-based photocatalysis stage further reduced COD to ∼5000 mg L^–1^ and BOD_5_ to ∼500 mg L^–1^, achieving overall removals of ∼95% COD and ∼90% BOD_5_, with turbidity below 200 NTU. Phytotoxicity assays indicated reduced toxicity after treatment, with a germination index of 90.04%. Techno-economic analysis showed improved profitability, with net income reaching 3.85 USD m^–3^ year^–1^, return on investment of 43.0%, payback period of 2.3 years, and net present value increasing from −0.15 to 18.57 USD m^–3^. These results demonstrate the efficiency and economic viability of the integrated system for dairy wastewater treatment.

## Graphical abstract

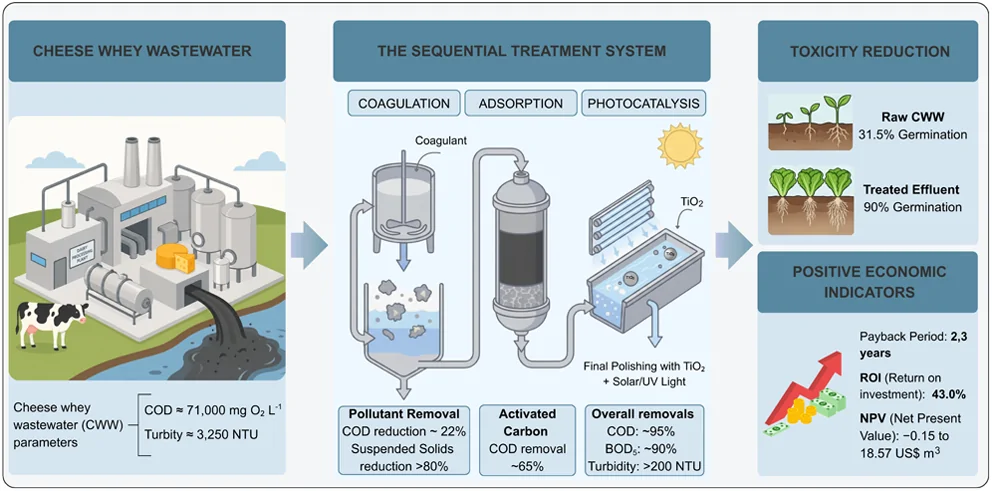

## 1 Introduction

The dairy industry is one of the largest food processing sectors worldwide, providing essential nutrients and supporting rural economies. However, it generates significant volumes of high-strength wastewater. Among the effluents produced, cheese whey constitutes the most abundant and environmentally challenging byproduct.^1^ Approximately 9–10 kg of whey is generated per kg of cheese. This effluent contains high concentrations of lactose, proteins, fats, and minerals, resulting in chemical oxygen demand (COD) and biochemical oxygen demand (BOD) that can exceed 10,000 mg O_2_ L^–1^. When discharged untreated, cheese whey rapidly depletes dissolved oxygen in receiving waters, promotes eutrophication, and emits strong odors, posing serious ecological and public health risks.^2^

Efficient and sustainable treatment of cheese whey remains a challenge.^2^ Biological methods, such as aerobic and anaerobic digestion, can reduce organic load but are often limited by the complex composition of whey, operational instability, and the need for precise control of environmental conditions.^3^ Physicochemical approaches, including membrane filtration and chemical oxidation, can achieve high removal efficiencies but require high energy input, specialized equipment, and chemical reagents, often generating secondary pollution or residual sludge. These limitations emphasize the need for strategies that are effective, environmentally compatible, and resource efficient.^4^

Integrated treatment systems combining complementary processes offer a promising solution for complex industrial effluents.^5^ For cheese whey wastewater, which contains both suspended and dissolved organics, sequential treatment can progressively remove different pollutant fractions while reducing energy and chemical demand. Such approaches align with the UN Sustainable Development Goals (SDGs), supporting responsible water management (SDG 6), waste reduction and circular resource use (SDG 12), and climate action through low-energy and renewable-driven processes (SDG 13).^5,6^

This study evaluates a sequential treatment system combining coagulation–flocculation, adsorption, and photocatalysis for the treatment of cheese whey wastewater, with emphasis on pollutant removal efficiency, process integration, and sustainability.^7^ The proposed approach integrates low-cost, biobased materials with solar-driven advanced oxidation to enhance treatment of high-strength dairy effluents.^5,8^ Unlike studies that evaluate each process independently, this work examines the synergistic effects and cumulative performance of the integrated treatment train, highlighting how each stage contributes to overall effluent quality improvement while reducing chemical and energy demands. In addition, the system supports circular economy principles by promoting waste valorization and minimizing sludge production.^9^ Overall, the study aims to demonstrate a scalable and environmentally sustainable alternative for dairy wastewater treatment, capable of meeting discharge requirements while contributing to more resource-efficient agro-industrial practices.

## 2 Material and Methods

### 2.1 Cheese Whey Wastewater Obtaining

Cheese whey wastewater (CWW) was obtained from a cheese factory in northern Paraná, Brazil. The whey was collected on-site during the curd-cutting stage of “Minas Frescal” cheese production. Immediately after collection, the material was passed through a cheesecloth to remove residual curd particles and other coarse suspended solids originating from the cheese-making process. After collection, it was filtered through cheesecloth to remove any suspended solids from the production process and subsequently stored at 4 ± 1 °C until its physicochemical properties were analyzed in triplicate.

### 2.2 Cheese Whey Characterization

During the remediation of CWW, the physicochemical parameters monitored included chemical oxygen demand (COD), biochemical oxygen demand (BOD_5_), turbidity (TUR), total suspended solids (SS), total solids (TS), and UV–vis spectral absorbance. These parameters served to evaluate the effectiveness of effluent decontamination.^7^ COD and BOD_5_ were measured according to the procedures recommended by the American Public Health Association.^10^ Turbidity was determined using a turbidimeter (model DLI-2500, Del Lab, Araraquara, Brazil) on samples diluted 1:50 with deionized water. Total solids and total dissolved solids were assessed via gravimetric analysis.^11^ UV–vis spectral absorbance was measured using a UV/vis spectrophotometer (model EEQ9011, Drawell, Chongqing, China) at the maximum absorption wavelength of 276 nm.

### 2.3 Coagulation/Flocculation Assays

Bench-scale coagulation/flocculation experiments were conducted using three 1 L rectangular jar testers (model MD013A, Eco Educacional, São José, Brazil) to evaluate the removal efficiency of COD, BOD_5_, TUR, SS, and TS. An anionic polyacrylamide (APA) coagulant (Lamirsa S.A., Barcelona, Spain) was prepared at a stock concentration of 1000 mg L^–1^. Reaction time and agitation speed were automatically controlled.

During the tests, CWW was dosed with APA at a concentration of 100 mg L^–1^ in 1 L of CWW, subjected to rapid mixing at 200 rpm for 1 min, followed by slow mixing at 48 rpm for 20 min to promote floc formation. The mixture was then allowed to settle for 2 h to enable decantation, after which the supernatant was carefully collected for physicochemical analysis and used in the subsequent adsorption step, according to previous works of the group.^7,12^ All experiments were performed in triplicate at a controlled temperature of 25 ± 2 °C.

### 2.4 Adsorption Assays

The adsorbent used in the tests was brewers’ spent grains activated carbon functionalized with phosphoric acid, synthesized following the method described by Castro et al.^13^ The adsorption experiments were carried out using a concentration of 10 g L^–1^ of the adsorbent with 50 mL of CWW supernatant obtained from the coagulation process. The samples were shaken for 12 h at 25 °C on an orbital shaker (TE-424, Tecnal, Piracicaba, Brazil). Afterward, the physicochemical parameters were analyzed, and the removal efficiency was determined. The supernatants were collected by filtration and subsequently used in the heterogeneous photocatalysis process.

### 2.5 Adsorbent Characterization

The activated carbon was characterized using scanning electron microscopy (SEM) coupled with energy dispersive spectroscopy (EDS) (VEGA3, TESCAN, Brno, Czech Republic) to obtain micrographs and determine their elemental composition. Nitrogen (N_2_) adsorption/desorption isotherms were measured using a sorption analyzer (NOVA 2000e, Quantachrome Instruments, Boynton Beach, United States). Fourier-transform infrared spectroscopy (FTIR, Spectrum Two, PerkinElmer, Shelton, United States) was also performed. The point of zero charge (PZC), or surface charge distribution, was determined by mixing 50 mg of the adsorbent with 50 mL of aqueous solution at various initial pH values (2–12), adjusted using 0.1 mol L^–1^ HCl or NaOH, and measuring the final pH after 24 h of equilibrium.^13^

### 2.6 Photocatalytic Assays

The supernatants from the adsorption experiments were subjected to photolysis and photocatalysis tests. The experimental setup consisted of a batch tank reactor, comprising a 250 mL cylindrical vessel (beaker) and a magnetic stirrer to ensure sample homogenization throughout the 24 h experimental period, and a 250 W mercury vapor Ourolux lamp without a protective bulb. For the photocatalysis experiments, 100 mL of the supernatant obtained from the adsorption step was placed in the reactor and exposed to artificial light irradiation, with 1 g of commercial TiO_2_ catalyst (anatase, 99.8%, Sigma-Aldrich, St. Louis, United States), calcined at 400 °C for 4 h. After the reaction period, samples from both assays were vacuum-filtered through cellulose ester membranes (47 mm diameter, 0.22 μm pore size) and analyzed for physicochemical parameters.^14^ The overall sequential treatment process, including CWW obtaining, coagulation/flocculation, adsorption, and photocatalysis, is summarized in Figure 1.

**Figure 1:**
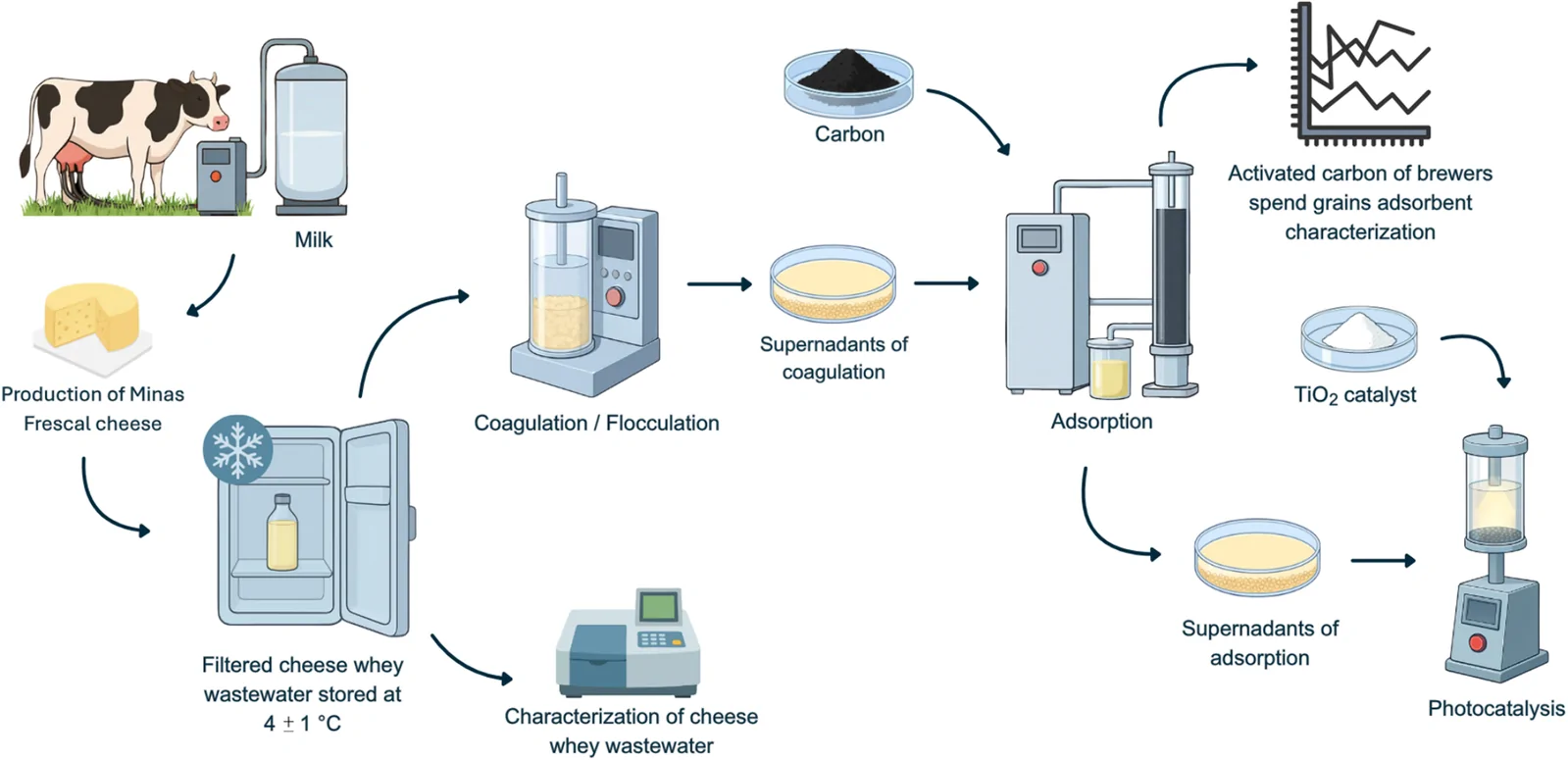
Schematic representation of sequential cheese whey wastewater treatment by coagulation/flocculation, adsorption, and photocatalysis.

### 2.7 Catalyst Characterization

The TiO_2_ catalyst was characterized using SEM/EDS, N_2_ adsorption–desorption isotherms, and PZC measurements, as described in Section 2.5. X-ray diffraction (XRD) analysis was performed using a diffractometer (XRD-7000, Shimadzu, Kyoto, Japan) equipped with a Cu Kα radiation source (λ = 1.54056 Å), operated at 30 kV and 20 mA, with a scanning rate of 2° (2θ) min^–1^; crystallinity index and graphitization degree were calculated as proposed by refs 15,16 .

### 2.8 Phytotoxicity Test

The phytotoxicity of CWW was evaluated before and after each treatment by determining the germination index (GI) and germination inhibition (GII). The assessment followed the method based on the postgerminative root growth of *Lactuca sativa* (iceberg lettuce). For each germination test, ten lettuce seeds were placed in Petri dishes lined with filter paper. Each dish received 5 mL of either raw or treated CWW. The experiment was conducted in five replicates, including a control group treated with distilled water.

All Petri dishes were incubated in the dark at 20 ± 2 °C for 7 days. Each day, approximately 0.1 mL of the corresponding test solution (CWW or distilled water for the control) was added to maintain moisture. Germination counts were recorded daily, and after 7 days, root lengths were measured. The GI and GII values were then calculated according to eqs 1 and 2.^17^

1 $$\mathrm{GI}=\left(\frac{G_{S}\times L_{S}}{G_{C}\times L_{C}}\right)\times 100$$

2 $$\mathrm{GII}=\left(\frac{G_{C}-G_{S}}{G_{C}}\right)\times 100$$

where GI (%) is the germination index percentage, *G*_S_ is the number of seeds that successfully germinated in the sample, *L*_S_ (mm) is the mean root length of seedlings in the sample, GII (%) is the inhibitory index percentage *G*_C_ is the number of seeds that successfully germinated in the control, and *L*_C_ (mm) is the mean root length of seedlings in the control.

### 2.9 Techno-economic Analysis for the Combined CWW Treatment

The economic analysis aimed to evaluate the financial feasibility of the three wastewater treatment configurations in this study: (i) coagulation/flocculation, (ii) coagulation/flocculation combined with adsorption, and (iii) coagulation/flocculation combined with adsorption and photocatalysis. All calculations were conducted on a unit basis of treated water (USD m^–3^) to allow for direct comparison among processes. The economic estimation of the proposed treatment scenarios was conducted using previously reported procedures and cost estimates.^18–20^ The economic evaluation was based on the optimal experimental conditions for the coagulation/flocculation, adsorption, and photocatalysis treatment processes, as well as their respective pollutant removal efficiencies when applied to CWW.

#### 2.9.1 Capital Investment Estimation

The capital investment was estimated for a treatment facility with a design capacity of 10 m^3^ day^–1^, representative of a small- to medium-scale industrial cheese whey wastewater treatment plant. Equipment was sized based on the process flow diagram and the optimal operating conditions determined experimentally. The same design capacity was adopted for all evaluated treatment configurations to ensure a consistent comparison of the economic indicators. It should be noted that the specific capital investment (USD m^–3^) depends on plant capacity. Larger-scale installations are expected to benefit from economies of scale, resulting in lower capital costs per unit volume of wastewater treated.

The total capital investment was divided into Fixed Capital Investment (FCI) and Working Capital Cost (WCC). The FCI included costs associated with land acquisition, civil construction (∼30%), piping systems, equipment purchase and installation (∼46%), power distribution, and engineering and supervision services. The WCC represents the capital required for the initial operation of the facility and was assumed to be equivalent to 15% of the FCI. The Total Capital Investment (TCI) was then calculated according to eq 3.^21^

3 TCI=FCI+WCC

Equipment costs were obtained from supplier quotations whenever available. In cases where quotations for the required equipment were not available, the costs were estimated by scaling from the reference size of similar equipment reported in the literature and shown in eq 4.^21^

4 $$C_{n}=C_{0}\times \left(\frac{F_{n}}{F_{0}}\right)\times {\left(\frac{h_{n}}{h_{0}}\right)}^{x}$$

where *C_n_* and *h_n_* are the cost and size, respectively, of the new equipment considered in this study; *C*_0_ and *h*_0_ are the cost and size, respectively, of the reference equipment reported in the literature; *x* is the cost-scaling exponent, which depends on the equipment type; and *f_n_* and *f*_0_ are the Marshall and Swift equipment cost indices for the base year (2026) and the original reference year, respectively.

#### 2.9.2 Operating Costs

Annual operating costs (AOC) were determined by summing the expenditures for chemicals and reagents, electricity, water, labor, maintenance, insurance, and other miscellaneous expenses. The operating costs associated with chemical consumables, including those used for pH adjustment and material cleaning/regeneration, as well as electricity consumption, were estimated according to eqs 5 and 6.

5 $$J_{c}=\Sigma \left(T_{c}\times R_{c}\right)$$

6 $$J_{E}=\Sigma \left(T_{E}\times R_{E}\right)$$

where *J*_c_ and *J*_E_ represent the costs of chemical consumption and electricity use, respectively (USD m^–3^ y^–1^); *R*_c_ (kg m^–3^ y^–1^) and *R*_E_ (kWh m^–3^ y^–1^) denote the corresponding chemical consumption and electricity usage rates; and *T*_C_ (USD kg^–1^) and *T*_E_ (USD kWh^–1^) are the respective unit prices of chemicals and electricity.

Annual operating costs included not only the purchase price of reagents but also the associated manufacturing, transportation, handling, and end-of-life management costs of the adsorbent and photocatalyst, which were incorporated into the commercial unit prices and miscellaneous operating expenses. These costs were aggregated in the economic analysis rather than reported individually. Although both activated carbon and TiO_2_ may be regenerated and reused in industrial practice, no regeneration cycles were assumed in the present assessment, resulting in a conservative estimate of operating costs. The values were derived from the process design data and current market prices (updated to 2026 USD).

#### 2.9.3 Revenue and Profit Calculation

The annual total revenue (*R*) was estimated from the sum of incomes associated with treated wastewater, sludge recovery, and water reuse. The economic benefits associated with the proposed treatment process were estimated based on pollution reduction and the potential for treated effluent reuse. For example, Dadebo et al.^19^ reported that the removal of 1 kg of COD could contribute approximately USD 0.15 to the project’s cash flow, reflecting the economic value of mitigating the environmental impacts associated with the discharge of highly polluted wastewater. Furthermore, the treated effluent should comply with the maximum permissible discharge limits established by local regulations and wastewater disposal guidelines, thereby enabling environmentally sound discharge or potential reuse. The economic benefit associated with sludge recovery was estimated considering the potential energetic valorization of the sludge generated during the coagulation/flocculation process through thermal conversion, as reported in previous studies.^7,22^ Moreover, on-site sludge utilization avoids transportation and disposal costs associated with third-party waste management services, further improving the economic performance of the treatment process. The gross profit (*L*) before depreciation and tax was calculated according to eq 7.^21^


7 L=R−AOC

#### 2.9.4 Depreciation, Taxes, and Cash Flow

Depreciation (*D*) was estimated using the straight-line method for a 10-year equipment lifetime, with a residual value equivalent to 5% of the FCI and determined according to eq 8.^21^

8 $$D=\frac{\mathrm{FCI}-S}{n}$$

where *S* is the salvage value and *n* = 10 years.

Earning before taxes (EBIT), net income (NI) after 25% tax rate applied to EBIT, and annual cash flow (CF), where calculated according to eqs 9, 10 and 11, respectively.^21^

9 EBIT=R−AOC−D

10 NI=0.75×EBIT

11 CF=NI+D

#### 2.9.5 Profitability Indicators

Three main indicators were used to evaluate economic feasibility, net present value (NPV) (eq 12), payback period (PBP) (eq 13), return on investment (ROI) (eq 14).^21^ The payback period represents the time required to recover the initial investment; in this case, shorter periods are more desirable. NPV measures profitability by comparing the present value of cash inflows with cash outflows over time, positive NPV values indicate that inflows exceed outflows, signifying a profitable venture. Finally, ROI serves as a key indicator from a business perspective, reflecting the overall efficiency and profitability of the investment.

12 $$\mathrm{NPC}=-\mathrm{TCI}+\sum_{t=1}^{10}\frac{{\mathrm{CF}}_{t}}{{\left(l+i\right)}^{t}}+\frac{S}{{\left(I+i\right)}^{10}}$$

where *i* = 10% is the annual discount rate.

13 $$\mathrm{PBP}=\frac{\mathrm{TCI}}{\mathrm{CF}}$$

A project was considered economically viable when NPV > 0, PBP ≤ 6 years, and ROI > 10%.^21^

14 $$\mathrm{ROI}=\frac{\mathrm{CF}}{\mathrm{TCI}}\times 100\%$$

#### 2.9.6 Economic Assumptions

The economic analysis was performed under a consistent set of financial and technical assumptions to ensure comparability among the evaluated treatment configurations. A project lifetime of 10 years was adopted, with all cash flows discounted at a rate of 10%, reflecting the expected opportunity cost of capital for medium-scale environmental technology investments. The corporate income tax rate was considered to be 25%, and a salvage value equivalent to 5% of the FCI was assumed at the end of the project horizon, representing the residual value of equipment and infrastructure.^23^ The working capital was estimated as 15% of the FCI to account for inventory, operational contingencies, and short-term liquidity needs. Furthermore, all financial data were expressed in purchasing power parity (PPP)-adjusted 2026 USD, assuming an annual inflation rate of 3%.^24^ These assumptions align with conventional methodologies for techno-economic assessment of wastewater treatment technologies and provide a standardized basis for comparing the performance and feasibility of the different treatment routes.^23–25^

### 2.10 Statistical Analysis

The results were analyzed using analysis of variance (ANOVA) to assess statistically significant factors and interaction effects between variables. Significant differences were tested using Tukey’s and Dunnet’s method (α ≤ 0.05).

## 3 Results and Discussion

### 3.1 Cheese Whey Wastewater Characteristics

In this study, the physicochemical characteristics of CWW prior to the treatment assays were determined, as summarized in Table 1. The CWW exhibited a slightly acidic pH (6.22), which can be attributed to the presence of organic acids such as lactic, fatty, and phenolic acids. This acidity strongly influences the coagulation/flocculation process, as charge neutralization is generally more effective under acidic to near-neutral conditions. The intrinsic variability in CWW composition underscores the importance of pH control to ensure reproducibility and optimal treatment performance.^26,27^

**Table 1:** Physicochemical Parameters of the CWW before Treatment

parameter	cheese whey wastewater^a^
pH	6.22 ± 0.25
turbidity (NTU)	3250.0 ± 740.0
total solids (g L^–1^)	65.22 ± 0.24
total dissolve solids (g L^–1.^)	37.42 ± 0.66
BOD_5_ (mg L^–1^)	4658.8 ± 45.55
COD (mg L^–1^)	70,985.1 ± 115.44
COD/BOD_5_	15.23

a Dry basis.

The measured COD and BOD_5_ were approximately 70,985.1 and 4658.8 mg L^–1^, respectively, resulting in a COD/BOD_5_ ratio of approximately 15.2. Since ratios above 4 indicate low biodegradability, these results confirm that CWW is poorly amenable to conventional biological treatment and requires advanced physicochemical processes for effective remediation.^2,28^

The CWW also showed high turbidity (3,250.0 NTU), total solids (65.22 g L^–1^), and total dissolved solids (37.42 g L^–1^). These elevated values, combined with the high organic load, highlight the complex and recalcitrant nature of this effluent, which poses a significant environmental concern if left untreated.^29,30^ The obtained composition is consistent with previously reported characteristics of CWW described by Castro et al.^2^ and Rivas et al.^9^

Given these characteristics, a sequential treatment strategy was employed to maximize pollutant removal. The process began with a coagulation–flocculation step using APA as a coagulant, aimed at removing suspended solids and reducing turbidity.^9^ Subsequently, an adsorption stage was performed using (H_3_PO_4_)-modified activated carbon, targeting the residual organic load and color removal through surface adsorption.^13^ Finally, a photocatalytic oxidation process employing TiO_2_ as a catalyst was applied to degrade the remaining recalcitrant organic compounds and further reduce COD levels.^7^

As reported by Rivas et al.,^26^ biological treatment alone is often insufficient and slow for CWW remediation. Thus, integrating eco-friendly physicochemical and advanced oxidation processes, such as coagulation/flocculation, adsorption, and photocatalysis, provides a sustainable and efficient alternative for treating this high-strength wastewater.^7^ In the present study, the removal efficiencies of COD, BOD_5_, turbidity, solids, and the reduction of UV–Vis spectral absorbance were evaluated across all three treatment stages.

### 3.2 Adsorbent and Catalysts Characterization

The physicochemical characterization of the adsorbent (A.C.) and photocatalyst (TiO_2_) is presented in Figure 2 and Table 2. The SEM micrograph of the A.C. (Figure 2a) reveals a highly porous and heterogeneous surface with a network of interconnected macropores and cavities, typical of carbon materials activated by phosphoric acid.^2,31^ Such a structure provides an extensive surface area and facilitates the diffusion of adsorbate molecules within the pore system during the adsorption process.^13,32^ In contrast, the SEM image of TiO_2_ (Figure 2b) displays agglomerated nanoparticles forming irregular clusters with quasi-spherical morphology, characteristic of the anatase phase.^33^ The nanoscale particle size increases the surface-to-volume ratio and provides a greater number of active sites, enhancing photocatalytic efficiency.^14,34^

**Figure 2:**
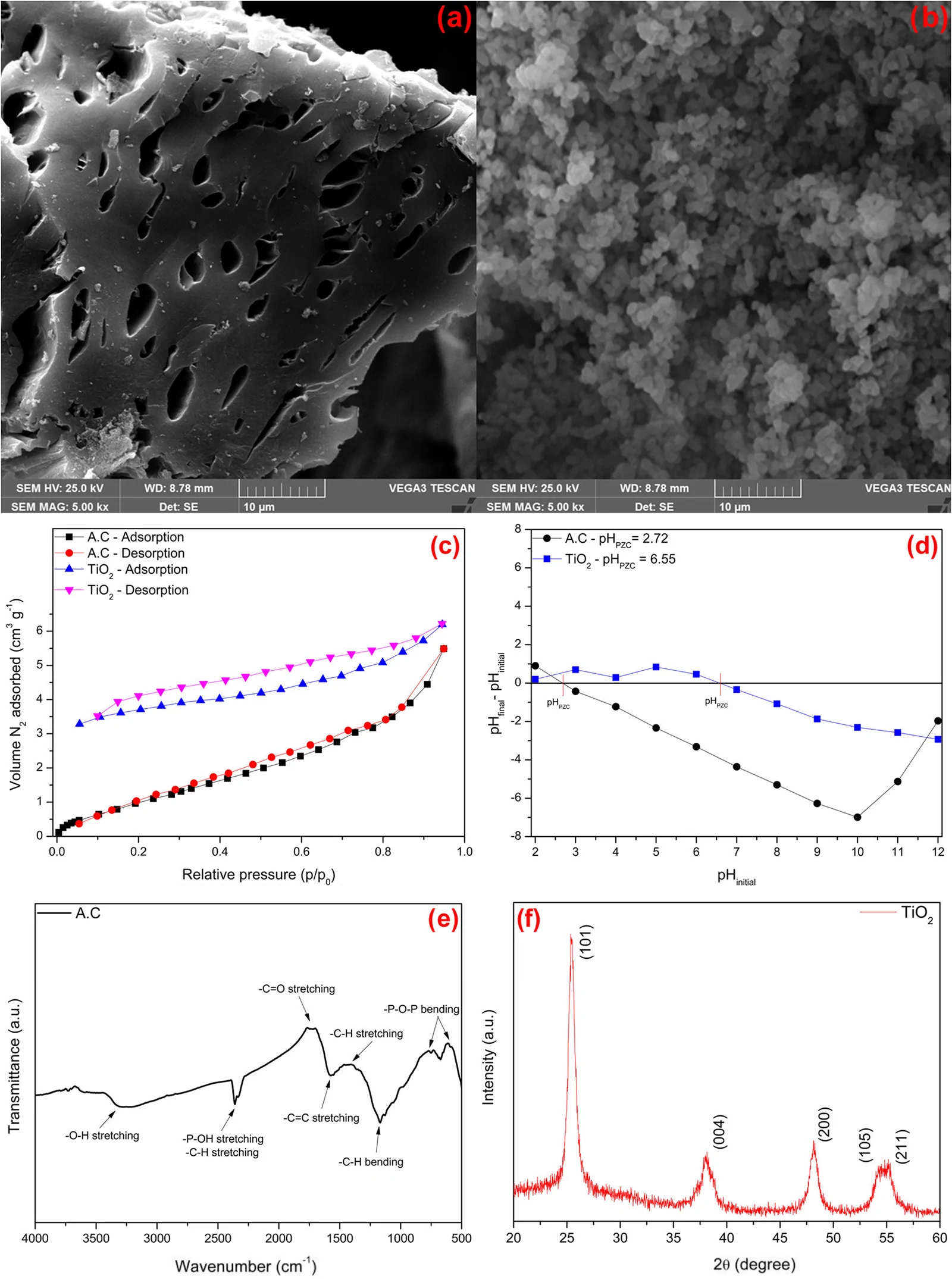
Characterization of the adsorbent and catalyst: (a) SEM micrograph of A.C. at 5000× magnification; (b) SEM micrograph of TiO_2_ at 5000× magnification; (c) N_2_ adsorption–desorption isotherms; (d) point of zero charge; (e) FTIR spectrum of A.C.; and (f) XRD pattern of TiO_2_.

**Table 2:** Textural, Structural, and Elemental Analysis of the Adsorbent and Catalyst

	N_2_ measurements at 77 K				
samples	*S*_0_ (m^2^ g^–1^)	*V*_p_ (cm^3^ g^–1^)	*d*_p_ (nm)	*d*_BJH_ (nm)	
A.C.	777.2	0.51	1.15	1.80	
TiO_2_	81.3	0.18	1.10	1.55	
Elemental analysis (%)					
Samples	C	P	N	**O**	**Ti**
A.C.	81.8	4.24	0.39	13.6	n.d.
TiO_2_	n.d.	n.d.	n.d.	40.1	59.9
Structural parameters					
Samples	Graphitization degree		Crystallinity index (%)		
A.C.	1.10		30.3		
TiO_2_			99.1		

The N_2_ adsorption–desorption isotherms (Figure 2c) and the corresponding textural parameters (Table 2) further support these observations. The A.C. isotherm resembles a Type I, with a steep initial uptake at low relative pressures.^35^ This pattern is characteristic of microporous materials, indicating that the material has a high volume of micropores, which is responsible for its high adsorption capacity at lower pressures.^36^ The specific surface area (*S*_0_ = 777.2 m^2^ g^–1^) and total pore volume (*V*_p_ = 0.51 cm^3^ g^–1^) are considerably higher than those of TiO_2_ (81.3 m^2^ g^–1^ and 0.18 cm^3^ g^–1^, respectively), confirming the highly porous nature of the carbon material. The mean pore diameter (1.15 nm) and BJH pore width (1.80 nm) suggest a predominantly microporous-to-mesoporous structure, which is advantageous for the adsorption of organic contaminants.^37^

In contrast, the TiO_2_ sample exhibited a Type II isotherm with a distinct H_2_-type hysteresis loop, characteristic of mesoporous materials composed of aggregated nanoparticles.^36^ This profile indicates the presence of mesopores formed by interparticle voids rather than a well-ordered pore network.^35^ The gradual increase in nitrogen uptake at higher relative pressures reflects capillary condensation within these mesopores, confirming the mesostructured nature of the catalyst.^37^ Although the specific surface area (81.3 m^2^ g^–1^) and total pore volume (0.18 cm^3^ g^–1^) are significantly lower than those of the activated carbon, these values are consistent with typical TiO_2_ photocatalysts synthesized by conventional methods. The mean pore diameter (1.10 nm) and BJH pore width (1.55 nm) indicate small mesopores and interparticle porosity that facilitate mass transfer and pollutant diffusion to the active sites. Such textural characteristics, combined with the high crystallinity of anatase TiO_2_, enhance light absorption and charge carrier transport, thereby improving photocatalytic efficiency.^33^

The point of zero charge (pH_pzc_) values (Figure 2d) were determined to be 2.72 for A.C. and 6.55 for TiO_2_. These parameters define the pH at which the surface carries no net charge. Below this point, the surface is positively charged, and above it, negatively charged.^7^ Given that the pH of the CWW was 6.22, A.C. would exhibit a negatively charged surface, favoring the adsorption of cationic species, whereas TiO_2_ would be slightly positively charged, promoting interactions with anionic pollutants to a lesser extent and cationic species to a limited degree.^13,33^ These surface charge characteristics are particularly relevant for understanding the adsorption–photocatalysis sequence, as they govern the electrostatic interactions between the materials and the contaminants during treatment.

Elemental analysis (Table 2) shows that A.C. consists mainly of carbon (81.8%) and oxygen (13.6%), with the presence of phosphorus (4.24%) and nitrogen (0.39%). The incorporation of phosphorus confirms successful chemical activation with H_3_PO_4_, introducing phosphate and hydroxyl surface groups, as corroborated by the FTIR spectrum (Figure 2e). The broad O–H stretching band around 3400 cm^–1^ corresponds to surface hydroxyls and adsorbed water, while peaks near 2920 cm^–1^ indicate C–H stretching of aliphatic groups.^38^ The signals observed at approximately 1100 cm^–1^ correspond to P–O–P and P–OH stretching, confirming the presence of phosphate functionalities that enhance the surface polarity and adsorption capacity.^39^ In contrast, TiO_2_ contains 59.9% Ti and 40.1% O, matching the theoretical stoichiometry of TiO_2_ and indicating high purity of the photocatalyst.^40^

The XRD pattern of TiO_2_ (Figure 2f) shows well-defined diffraction peaks at 25.3°, 37.8°, 48.0°, 53.9°, and 55.1°, corresponding to the (101), (004), (200), (105), and (211) planes of the anatase phase (JCPDS n° 21–1272).^41^ The absence of peaks related to rutile or brookite confirms the predominance of pure anatase TiO_2_. The sharpness of these peaks indicates high crystallinity (99.1%), consistent with the value reported in Table 2. Such high crystallinity enhances charge carrier mobility and minimizes electron–hole recombination, which is beneficial for photocatalytic performance.^42^ The A.C., in contrast, displays a low crystallinity index (30.3%) and a low graphitization degree (1.10), typical of amorphous carbon materials with disordered graphitic domains.^43^ This structural disorder increases the number of defect sites and contributes to enhanced adsorption through surface reactivity.^42,43^

Overall, the morphological, textural, and structural characterizations confirm that the activated carbon possesses a well-developed porous structure, enriched with oxygenated and phosphate functional groups, suitable for adsorption processes. TiO_2_ catalyst, on the other hand, exhibits nanostructured anatase morphology with high crystallinity and purity, ideal for photocatalytic oxidation. The complementary physicochemical properties of both materials justify their sequential use in the integrated treatment process, adsorption by A.C. followed by photocatalytic degradation using TiO_2_, maximizing pollutant removal efficiency from cheese whey wastewater.

### 3.3 Sequential Cheese Whey Wastewater Treatment

Figure 3 shows the effect of the combined treatment process on the removal of pollutants from CWW. The first stage, coagulation/flocculation, produced a noticeable improvement in the physicochemical characteristics of the effluent. COD (Figure 3a) decreased by approximately 22%, indicating the effective removal of a portion of the organic load, primarily associated with suspended and colloidal materials such as proteins, fats, and aggregated lactose.^26^ Similarly, BOD_5_ (Figure 3b) was reduced by around 37%, reflecting the partial elimination of biodegradable organic matter. Since coagulation/flocculation mainly targets particulate and colloidal components, a considerable fraction of dissolved organics remained in solution after this stage. Turbidity (Figure 3c) showed a pronounced reduction (30%), confirming the efficient aggregation and settling of fine suspended particles and protein–fat complexes typical of cheese whey wastewater.^30^ The suspended solids (Figure 3d) concentration also dropped substantially, with removal efficiencies exceeding 80%, demonstrating the strong capability of the coagulant–flocculant system to clarify the wastewater by separating the solid phase. In contrast, total solids (Figure 3e) decreased more moderately (about 26%), indicating that most of the dissolved fraction, such as salts, lactose, and low-molecular-weight organic compounds, remained in the supernatant. This partially clarified effluent was subsequently used in the next treatment step, adsorption with activated carbon, to target the remaining dissolved organic matter.^9^ The UV–Vis spectra support these observations, showing a consistent reduction in absorbance across the UV and visible regions after coagulation/flocculation, which is associated with the removal of aromatic and proteinaceous compounds.^7^

**Figure 3:**
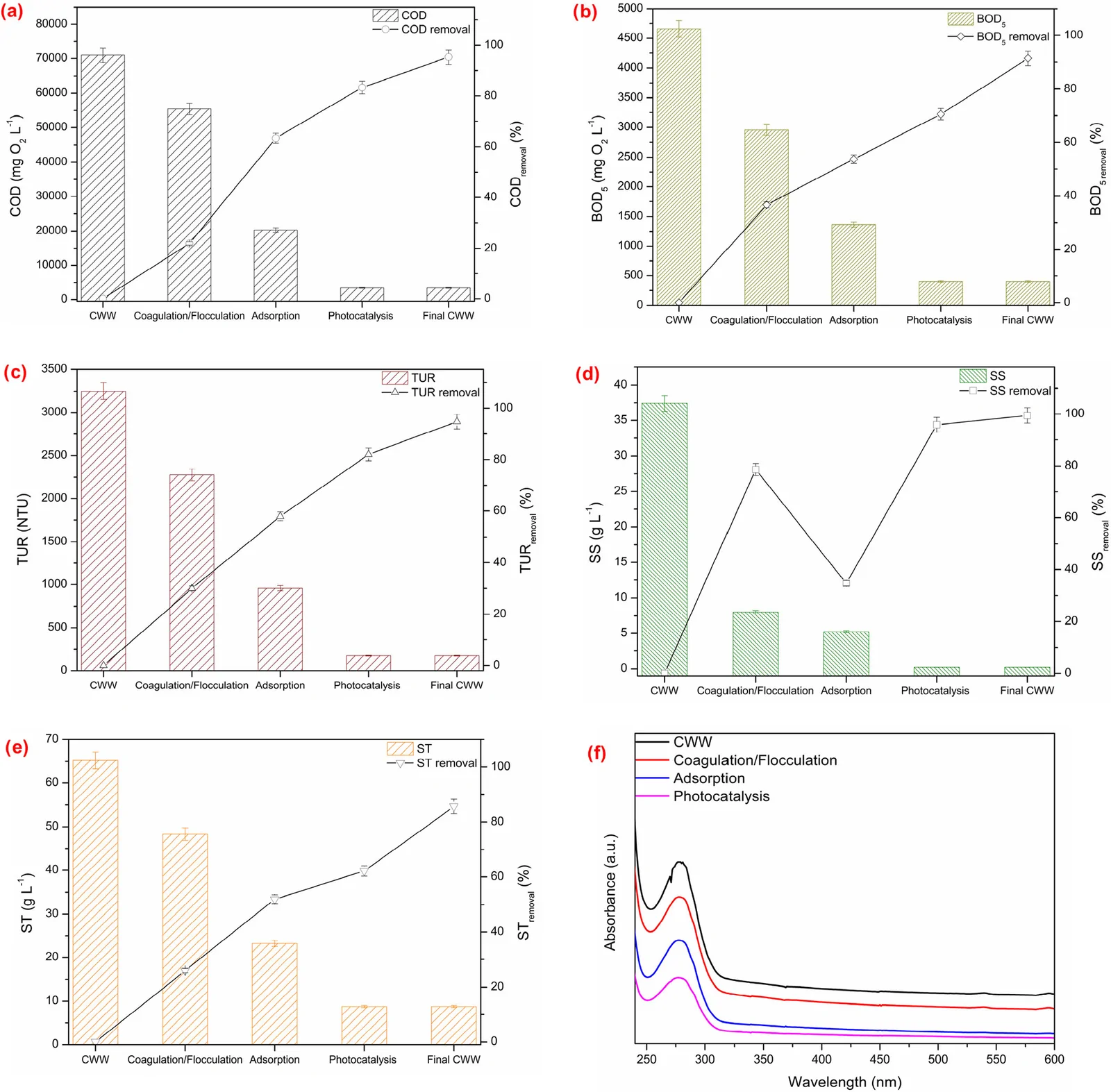
Physicochemical parameters after the combined treatment process for CWW removal: (a) COD removal; (b) BOD_5_ removal; (c) Turbidity removal; (d) Total dissolved solids removal; (e) Total solids removal; and (f) UV–vis spectra.

Overall, the coagulation/flocculation process served as an efficient pretreatment stage, reducing turbidity and particulate organic matter and producing a supernatant suitable for further purification through adsorption in the following step.

When compared with results reported in the literature, the removal efficiencies obtained in this study are consistent with previous findings for cheese whey wastewater treated by coagulation/flocculation. Rivas et al.^9^ evaluated FeSO_4_, Al_2_(SO_4_)_3_, and FeCl_3_ as coagulants for whey wastewater and achieved 40–60% reductions in COD and BOD_5_, depending on the coagulant type and dosage. In another study, Castro et al. (2026)^12^ used anionic polyacrylamide coagulant, reporting 78.7% BOD_5_, 95.7% turbidity, 36.9% lactose, 77.6% total dissolved solids, and 25.1% total solids reduction, values that closely match those obtained in the present work.

Therefore, the results obtained here align well with these studies, reinforcing the role of coagulation/flocculation as a suitable first step for cheese whey wastewater treatment. However, the persistence of dissolved organic matter in the supernatant, as observed both here and in prior works, highlights the necessity of subsequent treatment stages, such as adsorption, to achieve further purification and reduce residual organic load.

Figure 3 also presents the results obtained after the adsorption process using activated carbon as the second stage of the CWW treatment. The supernatant obtained from the coagulation/flocculation step was subjected to adsorption to further remove dissolved organic compounds and residual solids. A marked improvement in all physicochemical parameters was observed after adsorption, confirming the effectiveness of activated carbon in polishing the pretreated effluent.

A significant decrease in COD (Figure 3a) was achieved, reaching an overall removal of approximately 65% when both treatment steps are considered. This demonstrates that adsorption efficiently removed a large portion of the dissolved organic matter remaining after coagulation/flocculation.^2^ The BOD_5_ (Figure 3b) also showed a considerable reduction, with total removal approaching 55%, indicating that many biodegradable organic compounds were effectively captured on the activated carbon surface. This behavior aligns with the known affinity of A.C. for low-molecular-weight organic molecules, which are poorly removed by coagulation alone.^2,44^

Turbidity (Figure 3c) continued to decrease significantly after the adsorption step, with an overall removal of nearly 60%. This reduction is mainly attributed to the adsorption effects of the activated carbon pores, which trap fine colloidal particles and color-causing substances.^45^ SS (Figure 3d) also decreased, achieving a removal of approximately 40% relative to the remaining SS after coagulation/flocculation, confirming that the adsorption stage contributed to further clarification of the treated effluent.^7^ Similarly, ST (Figure 3e) removal reached about 55%, indicating that the adsorption process also captured part of the dissolved and colloidal solids that persisted after the first treatment step.^2^

The UV–Vis spectra (Figure 3f) support these findings. After adsorption, the absorbance curve showed a marked decrease across both the UV and visible ranges compared to the raw CWW and the coagulation/flocculation effluent. The strong absorbance reduction around 276 nm indicates the removal of aromatic compounds, proteins, and other chromophore organics, while the flattening of the curve suggests a substantial reduction in organic matter responsible for color and light absorption.

Overall, the adsorption process using activated carbon served as an effective polishing step, substantially improving effluent quality by targeting dissolved organic matter not removed during coagulation/flocculation. The combined treatment sequence thus achieved notable reductions in COD, BOD_5_, turbidity, and solids, producing a clarified and less polluted effluent.

These results are consistent with previous studies reporting the successful application of activated carbon for dairy or cheese whey wastewater polishing. For instance, Castro et al.^2^ found removal values for COD of approximately 90%, 70% for lactose and 50% for BOD_5_ when using activated carbon. Therefore, the present findings confirm that combining coagulation/flocculation with adsorption constitutes a promising and efficient strategy for the treatment of cheese whey wastewater, effectively addressing both suspended and dissolved pollutants.

Figure 3 presents the evolution of key physicochemical parameters throughout the treatment sequence, highlighting the effectiveness of the photocatalysis step as a polishing process and the overall improvement in final CWW quality.

As shown in Figure 3a, COD decreased sharply from the raw effluent (∼70,000 mg L^–1^) to approximately 20,000 mg L^–1^ after adsorption, followed by a drastic reduction to below 5,000 mg L^–1^ after photocatalysis. This corresponds to an overall removal efficiency approaching 95% in the final CWW. The significant drop during photocatalysis indicates the effective oxidative degradation of recalcitrant organic compounds that persist after physicochemical pretreatments.^7^

A similar trend is observed for BOD_5_ in Figure 3b. The initial value (∼4,600 mg L^–1^) was reduced progressively through coagulation/flocculation and adsorption, with a marked decline after photocatalysis to values below 500 mg L^–1^. The BOD_5_ removal efficiency exceeded 90% in the final effluent, suggesting not only a reduction in biodegradable organic matter but also an overall improvement in effluent stability and reduced microbial oxygen demand.^7^ Furthermore, the BOD_5_ removal efficiency far exceeded the minimum 60% reduction required for effluent discharge under the Brazilian CONAMA Resolution N° 430/2011, reinforcing the effectiveness of the multistage treatment process prior to effluent disposal.^46^

Turbidity removal (Figure 3c) followed a consistent decreasing pattern, from highly turbid raw CWW (∼3200 NTU) to values below 200 NTU after photocatalysis. The overall removal efficiency approached 90–95%, reflecting the combined effects of particle destabilization during coagulation and further elimination of fine colloids and light-absorbing substances during adsorption and photocatalysis. The low turbidity of the final effluent indicates improved clarity and reduced light scattering components.

In terms of SS, Figure 3d shows a substantial reduction from ∼37 g L^–1^ in raw CWW to near-zero levels after photocatalysis, corresponding to removal efficiencies exceeding 95%. While coagulation/flocculation achieved significant initial removal, fluctuations observed after adsorption suggest possible redistribution or partial solubilization of particulate matter. Nevertheless, photocatalysis effectively helped eliminated residual suspended solids, likely through oxidation and breakdown of particulate organic matter.^14^

TS, presented in Figure 3e, decreased from approximately 65 g L^–1^ in raw CWW to below 10 g L^–1^ after photocatalysis. Although the reduction trend is less steep compared to COD or turbidity, the overall removal efficiency still reached ∼85–90%. This indicates that while dissolved inorganic constituents may persist, a substantial fraction of total solids, particularly organic solids, was removed during treatment.^34^

The UV–Vis spectra (Figure 3f) further corroborate these findings. The raw CWW exhibited strong absorbance in the UV region (200–300 nm), indicative of aromatic compounds, conjugated systems, and other chromophore organic matter. A progressive decrease in absorbance intensity is observed after each treatment step, with the most pronounced reduction occurring after photocatalysis. The near-baseline spectrum of the photocatalyzed effluent confirms the effective degradation of UV-absorbing organic compounds, supporting the observed reductions in COD and turbidity.

Overall, Figure 3 demonstrates that while coagulation/flocculation and adsorption contribute significantly to the removal of bulk pollutants, photocatalysis plays a critical role in eliminating residual organic matter and improving effluent quality to near-complete detoxification levels.^2,12^ The integrated treatment approach thus ensures substantial reductions across all measured physicochemical parameters, producing a final effluent with markedly improved characteristics suitable for potential reuse or safe discharge.^7^

### 3.4 Phytotoxicity of Raw and Treated CWW

The phytotoxicity of CWW and its treated effluents was evaluated using the germination response of *Lactuca sativa*, a widely accepted bioindicator for assessing environmental toxicity. As illustrated in Figure 4 and quantified in Table 3, raw CWW exerts a strong inhibitory effect on seed germination and early seedling development, as evidenced by the low germination index (31.53%) and the high inhibition index (68.47%). This pronounced toxicity can be attributed to the high organic load characteristic of cheese whey effluents, including lactose, proteins, and residual organic acids, which collectively contribute to elevated chemical oxygen demand and may interfere with water uptake, enzymatic activity, and cellular metabolism during germination.^29^

**Figure 4:**
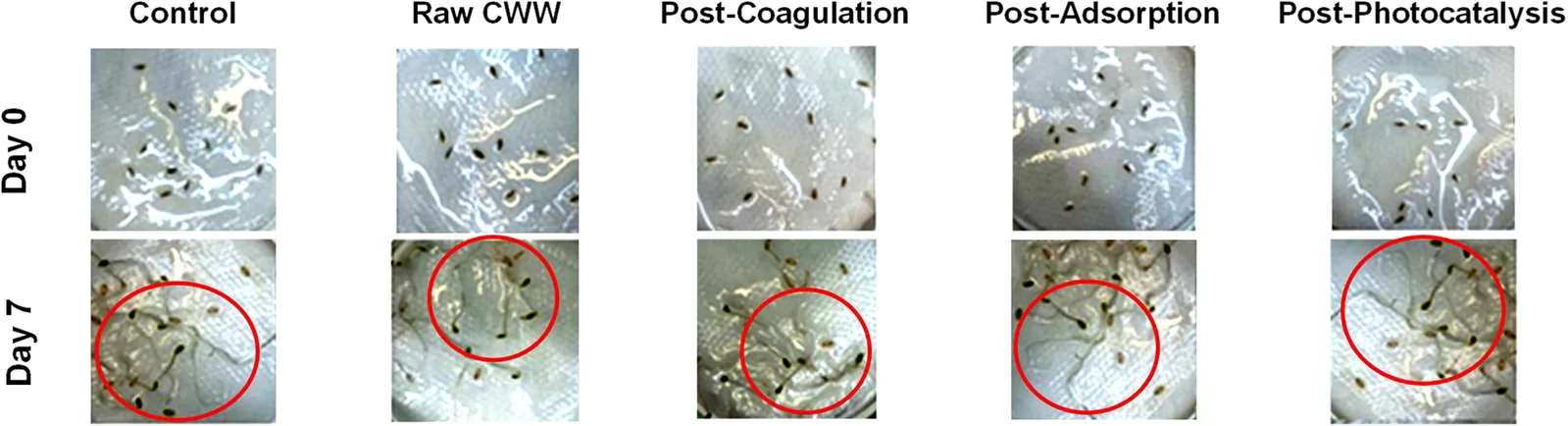
Germination of the *Lactuca sativa* (iceberg lettuce) exposed to raw and treated CWW. Legend: Red circles highlight representative regions with greater root development to facilitate visual comparison among the treatments.

**Table 3:** Germination Index and Inhibition Index of *Lactuca sativa* Exposed to Raw and Treated CWW^a^

	samples				
parameters	control	Raw CWW	post-coagulation	post-adsorption	post-photocatalysis
germination index (%)	99.12 ± 0.80^e^	31.53 ± 4.44^a^	48.98 ± 1.02^b^	73.21 ± 2.71^c^	90.04 ± 5.29^d^
inhibition index (%)	0.00 ± 0.00^a^	68.47 ± 5.02^e^	51.02 ± 2.55^d^	26.79 ± 3.33^c^	9.96 ± 4.44^b^

a Different lowercase letters indicate significant differences among the treatment steps according to Tukey’s test (*p* ≤ 0.05), whereas significant differences between the control and the treatment groups were determined using Dunnett’s test (*p* ≤ 0.05).

Following coagulation treatment, a moderate improvement in phytotoxicity was observed, with the germination index increasing to 48.98% and the inhibition index decreasing to 51.02%. This partial detoxification suggests that coagulation effectively removes a fraction of suspended and colloidal organic matter; however, a substantial proportion of dissolved and bioactive compounds likely remains in the treated effluent, maintaining a relatively high level of phytotoxicity.^12^ These findings indicate that coagulation alone is insufficient to achieve the level of detoxification required for safe environmental discharge or reuse.

A more pronounced reduction in toxicity was achieved after the adsorption step, where the germination index increased to 73.21% and the inhibition index decreased to 26.79%. This improvement highlights the effectiveness of adsorption processes in removing dissolved organic compounds and other inhibitory substances that are not eliminated during coagulation.^2^ The significant enhancement in seed germination suggests that adsorption plays a critical role in reducing the concentration of phytotoxic constituents, thereby improving the biological quality of the effluent.

The highest level of detoxification was observed following photocatalytic treatment, with the germination index reaching 90.04% and the inhibition index dropping to 9.96%, values approaching those of the control (99.12% and 0.00%, respectively). This substantial improvement indicates that photocatalysis is highly effective in degrading complex organic pollutants into simpler, less toxic molecules, likely through oxidative mechanisms that promote partial or complete mineralization.^47^ The near-complete recovery of germination capacity further corroborates the quantitative results, confirming the minimal residual toxicity of the treated effluent.

Overall, the progressive increase in germination index and corresponding decrease in inhibition index across the treatment sequence (raw CWW < coagulation < adsorption < photocatalysis), clearly demonstrate the cumulative effectiveness of the integrated treatment approach.^48^ The statistically significant differences observed among treatments (*p* ≤ 0.05) further confirm that each stage contributes meaningfully to toxicity reduction. From an environmental perspective, these findings indicate that while raw cheese whey wastewater poses a considerable risk to plant growth, advanced treatment, particularly when incorporating photocatalysis, can substantially mitigate phytotoxic effects and yield an effluent with characteristics suitable for potential agricultural reuse.^49^

### 3.5 Proposed Removal Mechanisms

The removal of pollutants from CWW in the proposed sequential treatment system involves a combination of physicochemical processes acting at different stages. Each unit operation, coagulation/flocculation, adsorption, and photocatalysis, contributes through distinct yet complementary mechanisms that enhance the overall treatment efficiency. Coagulation/flocculation primarily promotes the destabilization and aggregation of colloidal particles,^50^ adsorption enables the retention of dissolved organic compounds onto the surface of the adsorbent,^2^ and photocatalysis leads to the degradation of recalcitrant organic matter via reactive oxidative species.^33^ The integration of these processes results in a synergistic effect, improving the removal of organic load and turbidity.^47,51^ The proposed removal mechanisms for each treatment stage are schematically illustrated in Figure 5.

**Figure 5:**
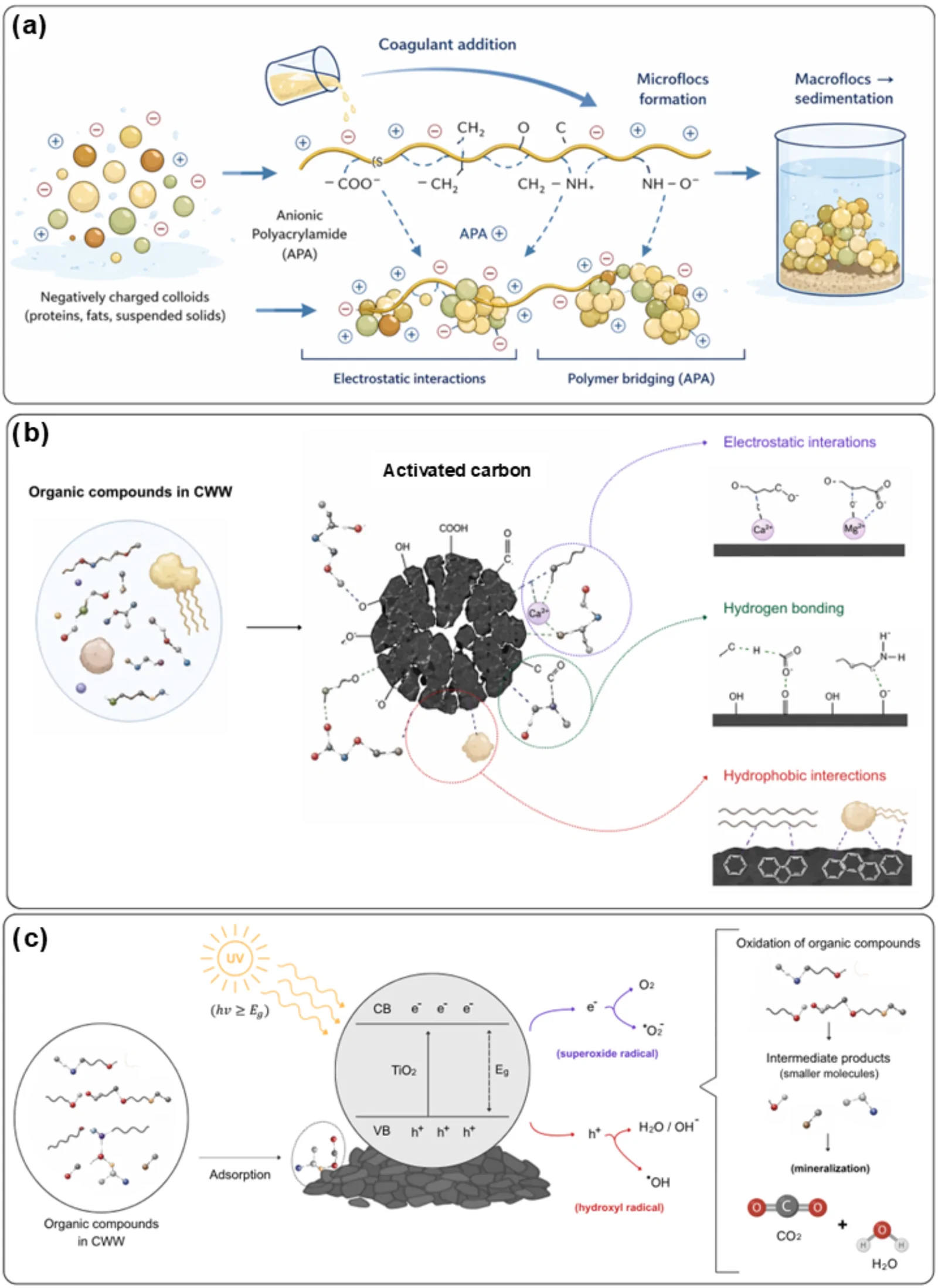
Proposed removal mechanisms for each step of the CWW treatment: (a) Coagulation/Flocculation, (b) Adsorption, (c) Photocatalysis.

The coagulation/flocculation mechanism of CWW using anionic polyacrylamide is illustrated in Figure 5a. Initially, CWW contains a high concentration of negatively charged colloidal particles, mainly proteins, fats, and suspended solids, which remain stable in solution due to electrostatic repulsion.^50^ Upon the addition of APA, two main mechanisms contribute to destabilization: charge neutralization and polymer bridging.^12^ Although APA is predominantly anionic, its functional groups can interact with positively charged sites or multivalent cations naturally present in the matrix, reducing the electrostatic repulsion between particles.^12^ This promotes particle aggregation through electrostatic interactions.^52^

Simultaneously, the long polymer chains of APA adsorb onto the surface of multiple particles, forming interparticle links known as polymer bridging. This mechanism enhances aggregation efficiency by connecting dispersed colloids into larger and more stable structures.^12^ As mixing conditions transition from rapid to slow agitation, particle collisions increase, favoring the formation of microflocs. Over time, these microflocs grow into larger and denser macroflocs, which exhibit improved settling properties.^12^ The increased size and mass of these aggregates allow their separation from the liquid phase by sedimentation, resulting in a clarified supernatant suitable for subsequent treatment processes.^52^

The adsorption mechanism of organic compounds present in CWW onto the activated carbon is illustrated in Figure 5b. The process is governed by multiple interactions occurring at the adsorbent surface, mainly electrostatic interactions, hydrogen bonding, and hydrophobic interactions, which act simultaneously to promote the removal of dissolved pollutants.^2^

Electrostatic interactions play an important role depending on the surface charge of the activated carbon and the ionic state of the pollutants.^2^ The presence of cations such as Ca^2+^ and Mg^2+^ in the CWW matrix can facilitate charge neutralization or act as bridging agents between negatively charged functional groups of organic molecules and the adsorbent surface. This interaction enhances the attraction of ionic species toward the carbon surface.^2^

Hydrogen bonding is another relevant mechanism, particularly due to the abundance of oxygen-containing functional groups (e.g., hydroxyl, carboxyl, and carbonyl groups) on both the adsorbent surface and the organic constituents of the wastewater.^45^ These interactions contribute to the stabilization of adsorbed molecules through specific donor–acceptor interactions, increasing adsorption capacity.^2^

In addition, hydrophobic interactions significantly contribute to the adsorption of nonpolar or weakly polar organic compounds.^40^ The aromatic and graphitic domains of the activated carbon provide hydrophobic regions that favor the association with aliphatic chains and hydrophobic moieties present in proteins, lipids, and other organic substances in CWW.^2^

The photocatalytic degradation of organic compounds present in CWW is illustrated in Figure 5c. This process is based on the activation of the TiO_2_ catalyst under UV irradiation, leading to the generation of highly reactive oxidative species responsible for the mineralization of organic pollutants.^33^

Upon irradiation with photons of sufficient energy (hν ≥ Eg), electrons in the valence band of TiO_2_ are excited to the conduction band, generating electron–hole pairs (e^–^/h^+^). These charge carriers initiate a series of redox reactions at the catalyst surface.^47^ The photogenerated holes (h^+^) react with water molecules or hydroxide ions adsorbed on the surface to produce hydroxyl radicals (•OH), while the excited electrons (e^–^) reduce dissolved oxygen to form superoxide radicals (•O_2_^–^).^33^

These reactive oxygen species (ROS), particularly hydroxyl radicals, exhibit strong oxidative potential and are capable of nonselectively attacking a wide range of organic compounds present in CWW, including residual proteins, lipids, and other dissolved organic matter. The oxidation process leads to the breakdown of complex molecules into smaller intermediates, which are further degraded into simpler end-products such as CO_2_ and H_2_O.^47^

Additionally, the adsorption of organic molecules onto the TiO_2_ surface enhances the efficiency of the photocatalytic process by increasing the contact between pollutants and reactive sites. The continuous generation of ROS, combined with surface-mediated reactions, promotes the progressive mineralization of organic contaminants.^33,47^

### 3.6 Techno-economic Evaluation for the Combined CWW Treatment

The techno-economic performance of the sequential treatment of CWW was evaluated considering capital investment, AOC, revenues, and key profitability indicators, in conjunction with the treatment efficiency previously discussed. The results (Table 4) clearly demonstrate that process intensification through adsorption and photocatalysis significantly enhances both technical performance and economic viability, despite the associated increase in costs.

**Table 4:** Cost Evaluation Analysis for the Sequential Treatment of 1 m^3^ of CWW

item	unit	coagulation/flocculation	coagulation/flocculation + adsorption	coagulation/flocculation + adsorption + photocatalysis
Initial investment cost				
Land usage	USD m^–3^	0.13	0.17	0.19
Structural and facility construction		1.80	2.85	2.93
Pipeline systems and components		0.47	0.67	0.73
Machinery purchase and installation		3.06	3.37	4.25
Power distribution and electrical installation		0.30	0.49	0.83
Engineering and supervision		0.44	0.61	0.69
Fixed capital investment (FCI)		6.20	8.16	9.62
Working capital cost (WCC)		0.93	1.22	1.44
Total capital investment (TCI)		7.13	9.38	11.06
Operating Expenses				
Chemicals and reagents	USD m^–3^ year^–1^	0.08	0.12	0.20
Anionic polyacrylamide coagulant		0.30	0.30	0.30
Activated carbon			0.55	0.55
TiO_2_ catalyst				1.27
Electricity		0.32	0.52	0.89
Water usage		0.02	0.02	0.03
Labor cost		0.05	0.10	0.15
Maintenance		0.13	0.17	0.25
Insurance		0.07	0.09	0.15
Other extra costs		0.10	0.24	0.53
Annual operational costs (AOC)		1.07	2.11	4.32
Profits				
Wastewater treatment	USD m^–3^ year^–1^	2.25	5.25	9.75
Sludge recover		0.10	0.10	0.10
Water reutilization				0.51
Total profits (revenues)		2.35	5.35	10.36
Economic Adjustments				
Depreciation (10 years)	USD m^–3^ year^–1^	0.59	0.77	0.91
EBIT (before taxes)		0.69	2.47	5.13
Taxes (25% EBIT)		0.17	0.62	1.28
Net income (after taxes)		0.52	1.85	3.85
Annual cash flow		1.11	2.62	4.76
Salvage value (5% FCI)	USD m^–3^	0.31	0.41	0.48
Profitability Criteria (10 years, 10%)				
Net present value (NPV)	USD m^–3^	–0.15	7.13	18.57
Payback period (PBP)	years	6.42	3.58	2.32
Return on investment (ROI)	%	15.6	27.9	43.0

TCI increases progressively from 7.13 to 11.06 USD m^–3^ when moving from coagulation/flocculation to the fully integrated system. This increase is mainly driven by machinery purchase and installation, which rises from 3.06 to 4.25 USD m^–3^, reflecting the addition of adsorption units and photocatalytic reactors. Structural and facility construction costs also increase (1.80 to 2.93 USD m^–3^), along with power distribution and electrical installation (0.30 to 0.83 USD m^–3^), the latter being particularly relevant due to the energy requirements of the photocatalytic process. In contrast, land usage remains low (0.13–0.19 USD m^–3^), indicating that the system is not constrained by spatial requirements and can be feasibly scaled up within existing industrial facilities.

The intensification of the treatment process leads to a substantial increase in AOC, from 1.07 to 4.32 USD m^–3^ year^–1^. This is primarily attributed to the introduction of activated carbon (0.55 USD m^–3^) and TiO_2_ catalyst (1.27 USD m^–3^), as well as increased electricity consumption (0.32 to 0.89 USD m^–3^). Additional contributions arise from higher labor, maintenance, and miscellaneous operational costs. However, these increases in operating expenses must be interpreted considering the significant improvement in treatment efficiency observed in Figure 3, where near-complete removal of COD, BOD_5_, turbidity, and solids was achieved after photocatalysis. The enhanced effluent quality not only ensures compliance with discharge standards but also enables water reuse, thereby generating additional economic value.

This is reflected in the strong increase in total revenues, which rose from 2.35 to 10.36 USD m^–3^ year^–1^. The major contribution comes from wastewater treatment benefits (2.25 to 9.75 USD m^–3^), directly associated with the high pollutant removal efficiencies, while the final integrated system also benefits from water reutilization (0.51 USD m^–3^). As a result, the net income (after taxes) increases markedly from 0.52 to 3.85 USD m^–3^ year^–1^, indicating a transition from a nearly nonprofitable process to a highly profitable operation. This trend is further supported by the increase in annual cash flow from 1.11 to 4.76 USD m^–3^, confirming the strong financial performance of the integrated system.

The profitability indicators provide additional evidence of economic feasibility. ROI increases from 15.6% for the standalone coagulation/flocculation process to 43.0% for the fully integrated system, demonstrating a substantial improvement in capital efficiency. Similarly, PBP decreases from 6.42 to 2.32 years, indicating that the integrated process can recover its initial investment within approximately half the time required for the conventional treatment. Importantly, the PBP of the integrated system is well below the assumed project lifetime (10 years), confirming its financial attractiveness. NPV further supports this conclusion, shifting from a negative value (−0.15 USD m^–3^) for the coagulation/flocculation process to positive values of 7.13 and 18.57 USD m^–3^ for the adsorption and photocatalysis-integrated systems, respectively. This transition from negative to positive NPV clearly indicates that only the combined processes are economically viable over the project lifespan.

The economic performance of the proposed integrated system is consistent with recent studies on combined treatment technologies for high-strength industrial wastewater, where process integration significantly enhances both treatment efficiency and economic viability. For instance, multistage systems combining coagulation, electrocoagulation, and adsorption of dairy wastewater have demonstrated high removal efficiencies and improved overall process performance due to synergistic effects between unit operations.^20^ These studies highlight that sequential treatment configurations outperform standalone processes by enabling stepwise removal of complex organic loads. Similarly, techno-economic analyses of advanced treatment technologies indicate that, although processes such as electrocoagulation or advanced oxidation may involve higher energy or operational demands, they offer advantages including compact footprint, rapid treatment, and improved water reclamation potential, which enhance their long-term economic feasibility.^50,51^ In this context, the present study demonstrates an even more pronounced economic benefit, with the net present value shifting from negative (−0.15 USD m^–3^) to strongly positive (18.57 USD m^–3^), a reduction in payback period to 2.32 years, and a return on investment reaching 43.0%.

These results confirm that the integration of adsorption and photocatalysis not only enhances pollutant removal efficiency and toxicity reduction but also ensures clear financial feasibility. Moreover, this work addresses a critical gap identified in the literature, where many studies remain limited to cost estimation without incorporating profitability indicators such as cash flow, ROI, or NPV, despite the recognized importance of these metrics for assessing real-world applicability.^53^

## 4 Limitations and Future Perspectives

Despite the promising technical and economic performance of the integrated coagulation/flocculation–adsorption–photocatalysis system for CWW treatment, several limitations should be acknowledged. First, although the combined process achieved near-complete removal of organic load and turbidity, the experiments were conducted at laboratory scale under controlled conditions. Scale-up to pilot and industrial levels may introduce challenges related to hydrodynamics, light penetration efficiency in photocatalytic reactors, and mass transfer limitations, which can affect overall treatment performance. In particular, the efficiency of photocatalysis is strongly dependent on irradiation uniformity and catalyst dispersion, which are difficult to maintain in large-scale systems. Although biological treatment is generally considered one of the most economical wastewater treatment technologies, its evaluation was beyond the scope of this study because the wastewater exhibited low biodegradability (COD/BOD_5_ ≈ 15). Future studies should investigate hybrid biological–physicochemical treatment systems and compare their technical and economic performance with the proposed process.

Second, the operational cost analysis revealed that the photocatalytic stage significantly increases annual operating costs, mainly due to TiO_2_ consumption and energy demand. Although the process remains economically viable, the reliance on chemical inputs and electrical energy may limit its application in regions with high energy costs or limited infrastructure. Additionally, catalyst recovery and reuse were not extensively addressed in this study, which may further impact long-term operational sustainability.

Another limitation lies in the composition variability of CWW. Cheese whey wastewater characteristics can fluctuate depending on production processes, seasonal variations, and raw material sources. While the proposed system demonstrated high efficiency for the tested effluent, its robustness under variable influent conditions requires further validation. Moreover, although toxicity reduction was achieved, a more comprehensive assessment of transformation byproducts formed during photocatalysis is necessary to ensure the absence of secondary pollution.

Although the proposed treatment system demonstrated high removal efficiencies for the evaluated parameters, nitrogen and phosphorus concentrations were not monitored in the present study. According to CONAMA Resolution N°. 430/2011, the monitoring of these nutrients may be required depending on the receiving water body and environmental licensing conditions. Furthermore, although the H_3_PO_4_-activated carbon was thoroughly washed after chemical activation to remove residual phosphate species, future studies should quantify total phosphorus in the treated effluent to confirm the long-term stability of the adsorbent and exclude any potential phosphorus leaching.

From an economic perspective, while key indicators such as NPV, ROI, and payback period confirmed the feasibility of the integrated system, the analysis was based on fixed assumptions (e.g., energy price, material costs, and project lifetime). Market fluctuations and regional economic conditions may influence these parameters, potentially affecting overall profitability. Therefore, sensitivity analyses considering variations in energy costs, catalyst price, and scale of operation would provide a more robust economic evaluation.

Future research should focus on optimizing the photocatalytic stage to reduce energy consumption, for example, using solar-driven systems or more efficient visible-light-responsive catalysts. The development of catalyst recovery and regeneration strategies is also essential to enhance process sustainability and reduce operating costs. In addition, integrating the proposed system with resource recovery approaches, such as nutrient recovery or biogas production from residual organics, could further improve economic performance and align with circular economy principles.

Furthermore, pilot-scale studies are necessary to validate process performance under real operating conditions and to assess long-term stability, maintenance requirements, and operational reliability. Advanced modeling and process control strategies could also be explored to optimize system performance and minimize energy and material consumption.

Overall, while the integrated treatment system demonstrates strong potential for effective and economically viable CWW treatment, addressing these limitations through process optimization, scale-up validation, and resource recovery integration will be essential for its successful implementation in industrial applications.

## 5 Conclusions

The sequential treatment system demonstrated high efficiency in the remediation of cheese whey wastewater, significantly improving its physicochemical characteristics. The initial coagulation/flocculation step reduced COD by ∼22%, BOD_5_ by ∼37%, turbidity by ∼30%, and removed more than 80% of suspended solids, confirming its effectiveness as a pretreatment stage. The subsequent adsorption process increased COD removal to ∼65% and BOD_5_ to ∼55%, highlighting the strong affinity of the activated carbon for dissolved organic matter. This performance was supported by adsorbent characterization, which revealed a porous structure and surface functional groups responsible for electrostatic interactions, hydrogen bonding, and hydrophobic effects.

The final photocatalytic treatment further reduced COD from ∼20,000 to <5,000 mg L^–1^ and BOD_5_ to <500 mg L^–1^, achieving overall removals of up to 95–100% for COD and >90% for BOD_5_, while turbidity decreased from ∼3200 to <200 NTU. In addition, phytotoxicity assays indicated a significant reduction in toxicity, with germination index increasing from 31% to 90%, demonstrating improved environmental safety. Techno-economic analysis showed that process intensification via adsorption and photocatalysis increased total costs (TCI = 7.13 to 11.06 USD m^–3^; AOC = 1.07 to 4.32 USD m^–3^ year^–1^) but enabled near-complete pollutant removal and boosted revenues (2.35 to 10.36 USD m^–3^ year^–1^). This resulted in a strong improvement in profitability, with net income reaching 3.85 USD m^–3^ year^–1^, return on investment of 43.0%, payback period of 2.32 years, and net present value increasing from – 0.15 to 18.57 USD m^–3^, supporting the feasibility of the proposed system.

Overall, the integration of these processes resulted in a strong synergistic effect, combining high treatment efficiency with sustainability and economic viability, making it a promising solution for the treatment of high-strength dairy effluents.
